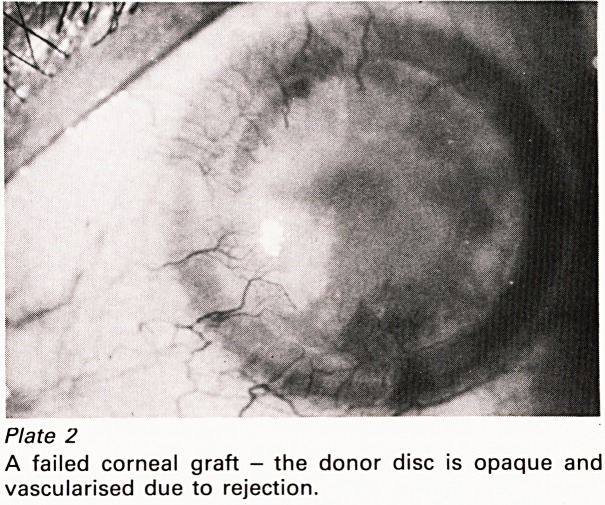# Corneal Transplantation in Bristol, 1970-80

**Published:** 1982

**Authors:** A. B. Tullo

**Affiliations:** Wellcome Trust Research Fellow Department of Ophthalmology, Bristol Eye Hospital, Lower Maudlin Street, BS1 2LX


### Corneal Transplantation in Bristol, 1970-80

**A. B. Tullo**
Wellcome Trust Research Fellow
Department of Ophthalmology, Bristol Eye Hospital, Lower Maudlin Street, BS1 2LX

### A DECADE OF CORNEAL TRANSPLANTATION IN BRISTOL

The transplantation of the cornea from a human eye to a human recipient (allografting) has been widely practiced since the 1940s as a method of treating corneal disease that is causing visual impairment. As with other complex surgical procedures, influences are constantly brought to bear on this technique which will modify and improve its already appreciable success rate, which now reaches 70-80%[1, 2] for some conditions. These influences are: improvements in microsurgical technique; advances in drug therapy including antivirals, and in particular anti-inflammatory and immuno-suppressive drugs; improved diagnosis and case selection aided by tissue typing of the potential recipient who has a vascularised cornea. The use of eye banks is commonplace in America[3], but in this country fresh donor material is used, usually within 24 hours of the death of the donor.

A review has been undertaken of all corneal grafts carried out in the Bristol Eye Hospital (BEH) during the years 1970-80. The results obtained in the first and second five year periods are compared, and discussed.

### METHODS

Cases were traced from the theatre record books. A limited amount of information could be obtained from all the notes. This included age and sex of the patient and diagnoses if known. Records of visual acuity pre- and post-operatively were frequently omitted in earlier notes. For this reason and because visual acuity is a limited measure of graft success (due to the concurrence of e.g. cataract, cystoid and senile maculopathy, and uncorrected astigmatism following grafting) attention was paid only to graft clarity to record success or failure.

Patients undergoing re-operation of a failed graft on one or more occasions, were considered as a separate diagnosis of 're-grafting'.

### RESULTS

The number of grafts performed during 1970-75 was 121, and during 1975-80 was 153. During the ten year period of the study, the 274 grafts were performed by 14 different surgeons. The number of grafts carried out by each surgeon varied from 2 to 71. The large majority were carried out under general anaesthetic.

*Diagram 1*

Corneal grafts at the BEH 1970-80

The total number of grafts may be subdivided into (a) penetrating and (b) lamellar grafts (partial thickness) (Diagram I). 31 (14%) of the penetrating grafts were combined with cataract extraction.

Patients receiving keratoplasty were: 48% female, and 52% male in 1970-75, and 54% female, and 46% male in 1975-80. The average age of women undergoing grafting was consistently higher than that of men (except in 1976). The percentage of patients below 50 years of age varied between 21% and 41%.

The diagnosis of the condition for which the graft was performed (including regrafting) is recorded in Table 1. The presence or absence of a clear graft was noted one month and one year post-operatively (Table 2). The maximum number of grafts performed on one eye was four. The maximum number of grafts performed for one patient was five. The commonest need for grafting in the two five-year periods was for a previous failed graft. Details of the commonest diagnosis leading to grafting are considered in Table 3.

### DISCUSSION

The figures obtained from this review confirm the clinical impression that herpes simplex keratitis,

**Table 1**

**Underlying Diagnosis in Patients having Corneal Grafts (expressed as percentage)**

| *Diagnosis* | *1970-75 (121 grafts)* | *1975-80 (153 grafts)* | *1970-80 (274 grafts)* |
|---|---|---|---|
| Regraft | 22.0 | 25.0 | 23.0 |
| Unknown | 15.0 | 16.0 | 15.5 |
| Herpes simplex keratitis | 15.0 | 13.0 | 14.0 |
| Dystrophies | 10.0 | 12.5 | 11.0 |
| Keratoconus | 8.0 | 11.0 | 9.0 |
| Pseudo-phakic* bullous keratopathy | 9.0 | 4.5 | 7.0 |
| Interstitial keratitis | 10.0 | 3.0 | 6.0 |
| Aphakic† bullous keratopathy | 1.5 | 3.0 | 2.0 |
| Others | 9.5 | 12.0 | 10.5 |

*following intraocular lens implantation
†following cataract extraction

**Table 2**

**Graft Clarity (as percentage of grafts for all diagnoses)**

| *Year* | *% clear at one month* | *Average* | *% clear at one year* | *Average* |
|---|---|---|---|---|
| 1970 | 36 | | 45 | |
| 1971 | 65 | | 42 | |
| 1972 | 40 | – 54% | 46 | – 45% |
| 1973 | 60 | | 35 | |
| 1974 | 70 | | 58 | |
| 1975 | 54 | | 38 | |
| 1976 | 65 | | 47 | |
| 1977 | 60 | – 67% | 53 | – 51% |
| 1978 | 74 | | 61 | |
| 1979 | 80 | | 56 | |

corneal dystrophies and keratoconus are the conditions that most commonly lead to corneal grafting, though they give no accurate indication as to the overall incidence of these conditions. The figures show that there is no significant shift amongst the list of conditions leading to grafting in the first and second half of the 1970s.

The percentage of cases with an undiagnosed corneal condition remained at 15-16% throughout the ten year period, suggesting that, while

**Table 3**

**Details of Patients Undergoing Grafts According to Diagnosis**

| *Diagnosis* | *no. of males* | *no. of females* | *Average age m.* | *Average age f.* | *% clear (1/12)* | *% clear (12/12)* |
|---|---|---|---|---|---|---|
| Herpes simplex keratitis | 24 | 14 | 55 | 55 | 63 | 54 |
| Dystrophies | 8 | 18 | 50 | 61 | 61 | 44 |
| Keratoconus | 15 | 9 | 27 | 41 | 78 | 78 |

Dystrophies were: Fuch's endothelial (52%)
Lattice (16%)
Unknown and others (19%)

documentation of corneal signs is undoubtedly better, there has been no major improvement in understanding the aetiology of some corneal conditions.

The success rate, as estimated at one year, of those grafts carried out in the last five years was 51% (Table 2), rising to 55% when therapeutic grafts (i.e. performed for perforation) are excluded. This seems a low rate when compared to the results of 70-80% now being achieved by single surgeons performing large numbers of this operation[1, 2, 4]. However, an American study which was comparable for its size and for the large numbers of surgeons involved, produced more compatible results[5]. An assessment was made of 203 grafts performed in 1972-74 by 40 different ophthalmologists, working in 15 different units served by the same eye bank. During 1975, 127 patients were also reviewed. In the first period the clarity rate at one year was 62% and in the second period was 53%. The study emphasised that the underlying diagnosis leading to grafting was the single most important factor influencing the prognosis.

Not only has the pattern of disease leading to grafting remained similar through the 1970s, but so have the average age of patients, the percentage of patients below 50 years and the male to female ratio. The demand for grafting has also remained steady despite the increasing number of referred patients from outside the catchment area of the Hospital. This suggests more careful case selection locally and elsewhere.

The elective use of local anaesthesia for surgery ceased after 1975 and thereafter appears to have been used only in patients who were unfit for general anaesthesia. The advantages of general anaesthesia for sophisticated microsurgery are obvious and a similar trend has occurred in cataract surgery. In 1970-71 18% of cataract extractions performed at the BEH were under general anaesthesia. In 1978-79 the figure had risen to 39%.

Post-operative follow-up, in particular for signs of rejection and raised intraocular pressure has improved. Despite an estimated incidence of a 10% rejection rate in uncomplicated cases[6], only one case of rejection was diagnosed prior to 1975. An indication of the improving follow-up measures is that in the second five-year period, 13 episodes of rejection have been documented, an overall incidence of 8% of cases. In five instances the rejection was reversed by treatment with steroids (38% success). The rate of rejection of grafts by the vascularised cornea is much higher[7] and prognosis can be improved by matching of the tissue types of donor and recipient[8] and an assessment of any prior sensitisation by pregnancy, blood transfusion and previous transplantation[9]. The number of matched grafts is at present restricted by logistical problems. It is hoped such problems can be circumvented as patients awaiting matched tissue occupy one third of the waiting list at the BEH. The proximity of the UK Transplant organisation at Southmead Hospital has aided this process and it is hoped that as more donor material becomes available, and that as more areas in the country list typed recipients, there will be a co-ordinated interchange of eyes, much in the same way that currently exists for kidneys.

The waiting list fluctuated between 26-51 averaging at 35 patients. It is clear that each time fewer grafts were performed than in the preceeding year the waiting list lengthened. The total number of 274 grafts during the ten years were performed at an average rate of one every two weeks, and with a relatively small increase in the rate of grafting the waiting list could be cleared, with the exception of patients awaiting matched donor material. The solution hinges on a controlled increase in the supply of donor material. This would not only reduce the waiting list, but if the supply was reliable, patients could be given a date for admission when listed and surgery could be carried out during routine lists. At present the collection of donor material and the location admission and operation of patients is done under emergency conditions.

The added advantage of a regular supply of donor eyes would mean that the criteria for acceptance, namely the age of donor and time from death to removal, could be reduced leading to improved quality of donor corneas. It is often difficult for medical staff to collect donor material during working hours and it seems worth while to

*Plate 1*
A successful graft – the pupil is visible through the clear donor disc and interrupted 10/0 Perlon sutures remain in situ 6 months after surgery.

*Plate 2*
A failed corneal graft – the donor disc is opaque and vascularised due to rejection.

concentrate on a few local sources with a high potential supply. Figures available for 1978 and 1979 show that the total number of deaths for the following hospitals and units are:

| | *1978* | *1979* |
|---|---|---|
| BRI | 598 | 708 |
| Radiotherapy Centre | 131 | 138 |
| ITU Frenchay | 83 | 63 |

There are very few medical contra-indications for using donor eyes. These include hepatitis B, syphilis, Creutzfeld-Jacob disease, and obvious eye disease. If only 10% of the potential local supply was used, 2 pairs of donor eyes would be made available for use every week, from the immediate vicinity of the BEH.

Evidence continues to emerge that it is the medical profession itself which is failing to make full use of the enormous potential supply of donor material for transplantation. One study revealed that while over half of a random sample of the population felt positively about giving eyes, kidneys and heart, only 1% of all deaths provide tissue for therapy, research and teaching[10]. That some neurosurgical units provide many more organs than others suggests that the supply reflects the attitude of the staff to the problems of procuring tissue for transplantation[11].

The chances of improving the supply of donor material are good in a city where the eye unit is part of a large hospital complex. It may even be possible to generate a surplus for research or export from the area as has been achieved with renal transplantation[11]. An improved supply is essential for the continuous revision and improvement of management and prognosis in patients undergoing corneal transplantation.

**ACKNOWLEDGEMENTS**

I am grateful to Professor D. L. Easty for advice and encouragement, to the Department of Medical Illustration, to the secretaries Mrs. M. Roach and Miss A. McSmythurs, and to Mr. R. Joomun (Statistics Officer) for the UBH mortality and BEH waiting list figures.

This project was funded by the British National Committee for the Prevention of Blindness.

**REFERENCES**

1. MAUMENEE, A. E. (1976) Recent advances in Corneal Transplantation. *Trans.Ophthalmol.Soc.* UK **96**, 462-70.
2. FOSTER, R. K. and FINE, M. (1971) Relation of donor age to success in penetrating keratoplasty. *Arch.Ophthalmol.* 85, 42-47.
3. POLACK, F. M., KUDO, T. and TAKAHASHI, G. H. (1968) Viability of human eye bank cornea. *Arch.Ophthalmol.* **79**, 205-11.
4. ABBOTT, R. L. and FORSTER, R. K. (1978) Determinants of graft clarity in penetrating keratoplasty. *Arch.Ophthalmol.* **97**, 1071-75.
5. FARGE, E. J. (1978) Results of penetrating keratoplasty over a four year period. *Ophthalmology* 85, 650-54.
6. POLACK, F. M. (1973) Corneal transplantation. *Invest.Ophthalmol.Vis.Sci.* **12**, 85.
7. GIBBS, D. C., BATCHELOR, J. R., WERB, A., SCHLESINGER, W., CASEY, T. A. (1974) The influence of tissue-type compatibility on the fate of full thickness corneal grafts. *Trans.Ophthalmol.Soc.* UK **94**, 101-26.
8. BATCHELOR, J. R., CASEY, T. A., WERB, A., GIBBS, D. C., PRASAD, S. S., LLOYD, D. F. and JAMES, A. (1976) *Lancet* **1**, 551-54.
9. STARK, W. J., TAYLOR, H. R., BIAS, W. B. and MAUMENEE, A. E., (1978) HLA and Keratoplasty. *Am.J.Ophthalmol.* **86**, 595-604.
10. FARGE, E. J. (1982) Human eye tissue for research. *Invest.Ophthalmol.Vis.Sci.* **22**, 128-29.
11. NICHOLLS, A. J., CATTO, G. R. D., EDWARD, N., ENGESET, J., LOGIE, J. R. C. and MACLEOD, M. (1980) Integrated dialysis and renal transplantation: small is beautiful. *Brit.Med.J.* **1**, 1516-17.

## Figures and Tables

**Diagram 1 f1:**
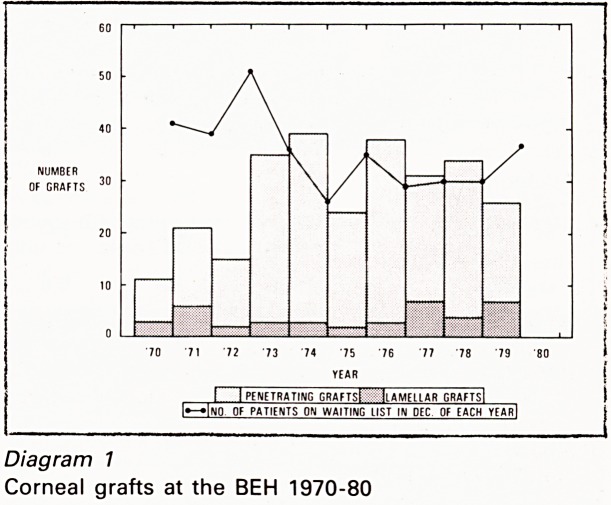


**Plate 1 f2:**
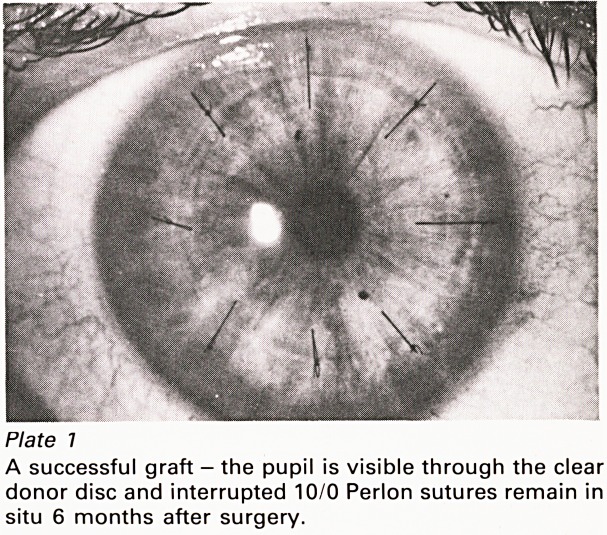


**Plate 2 f3:**